# What influences patients with Osteoarthritis to consult their GP about their symptoms? A narrative review

**DOI:** 10.1186/1471-2296-14-195

**Published:** 2013-12-20

**Authors:** Zoe Paskins, Tom Sanders, Andrew B Hassell

**Affiliations:** 1 Arthritis Research UK Primary Care Centre, Keele University, Keele ST5 5BG, UK; 2 School of Medicine, Keele University, Keele ST5 5BG, UK

**Keywords:** Osteoarthritis, Primary health care, General practitioners, Utilisation

## Abstract

**Background:**

Osteoarthritis (OA) is a common cause of disability and consultation with a GP. However research suggests the majority of sufferers choose not to consult their GP regarding their symptoms. Understanding the reasons for consulting is central to optimising patient outcomes. This review aims to summarise existing literature to identify what influences patients with OA to consult their GP.

**Methods:**

Due to the diversity of both qualitative and quantitative research that has addressed this research question a narrative review of literature has been conducted, backed up by a systematic literature search.

**Results:**

Nineteen papers were identified describing influences on consulting behaviour in patients with likely OA. Health beliefs, such as perceiving OA as an inevitable part of older age about which nothing can be done, in addition to perceiving a negative attitude of the GP, are disincentives to consulting. Severity of pain and disruption of daily activities are important influences towards consultation. Social issues such as the availability of support networks are also likely to be influential. Evidence is lacking about the impact of multi-morbidity on consulting behaviour.

**Conclusions:**

Pain and disruption of activities appear to push towards consulting and negative attitudes regarding OA (from either the patient or GP) appear to be disincentives to consulting. Findings are limited by estimates of consultation frequency and research involving observation of consultations may improve understanding of these issues. Specifically, further research may address how pain and disrupted function are addressed and if negative attitudes are evident in the consultation.

## Background

Osteoarthritis (OA) is a leading cause of pain and morbidity, and globally, is the fastest increasing cause of disability [1]. Despite this, a number of studies report low rates of consulting with a General Practitioner (GP) [2-6], with 17% of patients with OA consulting annually [3] and over 50% of those with severe pain not consulting over an 18 month period, in the UK [2].

Understanding the precursors or antecedents to the consultation is important for researchers and clinicians who aim to enhance the care of patients with osteoarthritis in primary care for two reasons. Firstly, the reasons for consultation are intertwined with an individual’s ideas, concerns and expectations [7], and their agenda for the consultation. Increasingly, the events in the consultation have become a focus of interest for researchers. Any consultation intervention to enhance care of OA needs to address patients’ agendas. Secondly, the reasons for seeking or using healthcare go hand in hand with reasons for not consulting, and hence understanding the drivers to consultation may further understanding of why many OA sufferers choose not to consult their doctor, described as the 'iceberg of morbidity’ [8].

A number of studies have evaluated the influences on patients’ decision making around seeking healthcare for OA symptoms. This review aims to summarise what is currently understood about why patients with OA consult their GP, and was conducted within a wider programme of research aiming to enhance the care of patients with OA in primary care.

## Methods

An initial literature search, performed as a scoping exercise identified relevant research using a wide range of both quantitative and qualitative research methods. Due to the diversity of studies, a narrative review was therefore felt to be most appropriate to confer the flexibility needed to review the relevant literature. A narrative review is described as a 'first generation 'traditional’ literature review’; narrative reviews have a useful place for identifying themes and gaps in the literature and for informing direction of further research [9]. This review has been enhanced by a systematic literature search; combining narrative and systematic methods has value in enhancing transparency and rigour of narrative reviews [9]. Studies have been identified by searching relevant databases (Medline, CINAHL, Psychinfo and Google scholar), in addition to reference checking, manual searching of the contents page of relevant journals and recommendations from experts. The search was divided under three headings: population (patients with osteoarthritis); setting (primary care) and consulting (consultations between GPs and patients). No search terms were used to limit the 'influences’ on consulting to avoid the risk of excluding relevant papers. Search terms used are shown in Table 1.

**Table 1:** Search terms used to identify influences on consulting behaviours in OA

**Setting: primary care**	**Population: patients with OA**	**Consulting**
Primary health care	Osteoarthritis	Consult* AND behavio*
GP OR general practitioners	Osteoarthritis, knee	Consult* AND frequency
Family physicians	Osteoarthritis, hip	Consult* and prevalence
Family practice	Arthritis	Seek
General practice		Visit
		Utili*ation

Notes

1. Setting and Population terms searched as MeSH headings.

2. Consulting terms searched as keywords.

3. Results within columns combined with OR operator, results across columns combined with AND operator.

Inclusion of papers was deliberately not restricted. The aim was to include the broadest possible sample of papers, to determine all possible influences on consulting. Studies reporting consulting behaviour that have sampled older adults with joint pain, in contrast to clinically or radiologically diagnosed osteoarthritis have been included with two assumptions: that the factors affecting consulting rates are likely to be similar, and that patients with osteoarthritis are likely to account for the majority of respondents. Similarly, no exclusions were made on the basis of study design or patient population studied with the assumption that all studies may further understanding about consulting behaviours. 'Primary care consultations’ were defined as consultations between a GP and a patient for the purpose of this review, with consultations with other members of the primary care team excluded. To appraise the evidence, no single tool was appropriate for the diverse range of studies included. Particular attention has been paid to the characteristics of the sample and setting which may affect the transferability of the findings.

In a number of papers identified in this review, the Andersen- Newman model was used to describe influences on consulting [2,10-13]. This framework is used to describe factors that influence healthcare utilisation and is divided into three areas [14]:

1. Predisposing factors, the social and cultural characteristics of a person (including factors that may have existed prior to illness)

2. Enabling factors, the logistical issues affecting accessing care

3. Need factors, which are the most immediate cause for seeking healthcare and are usually related to the illness itself.

The influences on consulting behaviour have been classified under these headings in the results and discussion, in order to provide a framework for the narrative review and to organise discussion of similar themes.

## Results and discussion

Table 2 summarises the papers identified with respect to their methodology and the influences on consulting behaviour identified, as classified by the Andersen Newman model. There is some overlap in the scope of the three categories; some authors have already classified the influences they measured using the model [2], and in these instances the authors' own classification has been applied, leading to certain themes (such as health beliefs) appearing in more than one column. Sixteen papers evaluated need factors, 15 evaluated predisposing factors and ten papers evaluated enabling factors. Individual themes are discussed below.

**Table 2:** Summary of papers identified exploring influences on consulting behaviours in OA

**First author, year**	**Population studied Age (OA or joint pain (JP)) Country of origin**	**Methodology**			**Influences evaluated**		
		**Quantitative**	**Qualitative**	**Mixed**	**Predisposing**	**Need**	**Enabling**
**Arthritis Care (2012) [**[23]**]**	OA, UK	•			Age	Pain	Health beliefs^*^
**Bedson (2007) [**[2]**]**	≥ 50, JP (knee),	•			Gender	Pain	
	UK				Occupational class	Disability	
					Educational attainment	Comorbidities	
					Marital status		
					Social network		
					Depression		
**Coxon (2012) [**[20]**]**	≥ 50, JP (hand, knee or hip)	•			Health beliefs	Pain	
	UK					Disruption of daily activities	
						Comorbidities	
**Cronan (1995) [**[11]**]**	≥ 60, OA (defined by symptoms)	•			Depression	Health status (as measured by arthritis impact)	Previous healthcare use
	USA				Self-efficacy		Social support
					Age		
					Gender		
**Dieppe (1999) [**[13]**]**	Literature review and consensus techniques with health professionals			•	Health beliefs	Functional status	Previous experience of healthcare
							Family beliefs and expectations
**Grime (2010) [**[24]**]**	≥ 50, OA or JP (self-report), UK		•		Age	Onset and severity of pain	
**Hill (2007) [**[4]**]**	≥ 50, OA hand (self-report), UK	•			Health beliefs		
**Hoogeboom (2012) [**[12]**]**	OA, Netherlands	•			Age	Pain	Previous healthcare use
					Gender		
					Ethnicity		
**Jinks (2007) [**[18]**]**	≥ 50, JP (knee), UK			•	Health beliefs	Severity of pain	
**Jordan (2006) [**[10]**]**	≥ 50, JP (knee), UK	•			Age	Widespread pain	Practice registered with education
					Gender	Frequent consulter	Cohabiting
					Anxiety	Pain duration	Previous use of GP
					Depression	Bilateral symptoms	
						Previous injury	
**Linsell (2005) [**[6]**]**	≥ 65, JP (hip and knee), UK	•				Joint affected	
**McHugh (2007) [**[22]**]**	OA patients awaiting joint replacement, UK		•			Pains severity	
						Visits to GP with other problems	
**Mitchell (2006) [**[16]**]**	≥ 50, JP (knee), UK	•			Age	Severity of pain	Urban GP practice
					Gender		
					Health beliefs		
**Rao (1997) [**[15]**]**	≥ 18, self-reported arthritis	•			Age	Activity and work limitation	
	USA				Gender	Area of residence	
					Ethnicity	Doctor visits for other Health problems	Income
					Overweight		Health insurance
**Rosemann (2007) [**[21]**]**	OA, Germany	•			Age	Comorbidities	Previous healthcare use
					Gender	Number of prescriptions	Marital status
					Obesity	Pain severity	
						Physical limitation	
**Sanders (2004) [**[19]**]**	≥ 51, OA (self report) UK		•		Age		Previous use of healthcare
					Health beliefs		
**Schellevis (1994) [**[27]**]**	age not stated, OA, Netherlands	•				Comorbidities	
**Thorstensson (2009) [**[17]**]**	≥ 35, JP (hip or knee), UK	•			Obesity	Comorbidity	Living in urban area
					Age	Which joint affected	Living in deprived area
					Gender	Mobility problem	
					Depression	Pain severity	
**Watts (2011c) [**[25]**]**	JP, UK		•		Age	Site of pain	

^*^Classifed by Bedson et al. as enabling.

### Predisposing factors

#### Gender, age and body mass index (BMI)

A prospective study of consulting behaviour of older adults with knee pain demonstrated that female gender was a significant predictor of a new episode of consultation [10] and this finding is replicated in an American study of patients with self-reported arthritis [15]. However, four other UK studies do not report any influence of gender on consulting [2,4,16,17].

Similarly, there does not appear to be a clear influence of age on consulting. Jordan et al. [10] found a modest increase in incidence of consultations in patients aged 65-74, although this lost significance when adjusted for other variables. In a postal survey of patients with self-reported hand problems, those over 70 were less likely to have consulted their GP about their hands in the last 12 months when adjusted for other significant factors [4]. However, two American studies report that younger patients with arthritis are less likely to consult [11,15].

Jordan et al. [10] and Thorstensson et al. [17] have both demonstrated a modest association between obesity and likelihood of future consultation about knee pain; in the former study this lost significance when corrected for other factors. Rao et al. [15] also reported an association between being overweight and self-reported consultation rates.

#### Health beliefs

The influence of health beliefs on consulting was considered in both qualitative work looking at individuals’ perspectives and in quantitative population studies. Qualitative research reveals the nature of the beliefs and quantitative studies are useful for establishing the effect of the beliefs on behaviour. Prevalent disorders, such as joint pain, in the elderly may be regarded as less serious or as a normal consequence of ageing [13,18], and therefore not necessarily a symptom of illness [13,18,19]. It has been suggested that by not consulting, patients may seek to maintain a 'healthy’ identity [18].

Some patients hold the belief that OA is not a treatable condition, that 'nothing can be done’, and this may have been reinforced by previous visits to GPs [18,19]. Coxon et al. [20] describe results from a choice based conjoint analysis study where the perceived attitude of the GP was an important determinant in deciding whether or not to consult the GP. This was second only to restriction of activities, and found to be more significant than other health problems and episodes of severe pain.

Population studies demonstrated significant effects of health beliefs on consulting. Mitchell et al. [16] reported that participants who held beliefs that their (knee) pain would have a permanent effect and that it affected the way they were viewed by others, were more likely to consult when corrected for other significant variables. A larger postal survey of patients with self-reported hand symptoms (including OA) also demonstrated illness perceptions associated with consulting, including believing the hand problem was permanent/ would last a long time; believing that treatment could control symptoms and reporting more severe perceived consequences of hand pain. This study also demonstrated frustration and 'emotional representations’, which included statements about anger, were associated with consulting a GP [4]. Positive perceived general health status has also been associated with non-consulting for patients with self-reported arthritis [11,15].

Self-efficacy has been shown to inversely correlate with total healthcare visits in patients with OA in another study, and was the psychological variable which best predicted healthcare use [11].

#### Depression

Depression is an important condition that may be a barrier to consulting but again, the evidence here is somewhat contradictory. Consulters with severe knee pain in a study reported by Jordan et al. [10] were significantly less depressed than non-consulters. However, in contrast, the total number of GP visits by patients with OA has been reported to correlate positively with depression scores [21]. Thorstensson et al. [17] did not demonstrate an association between anxiety and depression and consulting in a population with self-reported hip and knee pain; however, in this study the population were aged 35 and over and there may have been a significant proportion of participants who did not have OA. The relationship between anxiety and depression and consulting may be mediated by health beliefs; Hill et al. [4] reported associations between anxiety and depression and certain health beliefs (e.g. frustration), but unfortunately did not investigate for relationships between anxiety and depression directly with consulting behaviour.

In summary, the pre-disposing factors which appear to have the clearest association with consulting are health beliefs. Holding beliefs that OA can be treated successfully and perceiving severe consequences of pain have been associated with consulting in population studies, whereas believing OA is a 'normal’ consequence of ageing or that the GP may have a negative attitude towards OA are described as disincentives to consulting. Anger, frustration and depression may also be associated with consulting but the evidence here, particularly for depression is less clear.

### Enabling factors

#### Previous use of healthcare

Jordan et al. [10] reported that a previous knee injury was one of only three predictors of consulting with knee pain that remained significant when adjusting for all other variables. Jordan et al. attribute this to previous contact with the GP and knowledge of the healthcare system. In this study, having previously used non-GP services was also a significant predictor of seeking healthcare in the participants with severe pain. A Dutch study also reported previous healthcare use as a predictor of consulting with joint pain [12]. However, a previous visit to the GP regarding joint pain may be a barrier to further consultation if the patient has encountered a negative attitude from the GP; patients have reported hiding their symptoms in this context [22]. Patients also reported very few consultations with GPs while on the waiting list for joint replacement surgery, feeling they were 'under a specialist’ and so joint symptoms were no longer the remit of the GP [22].

#### Cohabiting and social networks

Rosemann et al. [21] reported that living alone was a predictor of number of GP attendances (all reasons) and living alone was also a weak predictor of consulting for knee pain in the study by Jordan et al. [10]. This may be explained by lack of a social network although no studies have examined this directly.

#### Area of residence

Living in an urban area has been reported as a strong predictor of consulting with hip and/ or knee pain, whereas deprivation scores were not significantly related to consultation rates [17]. In contrast, Mitchell et al. [16] reported social domain score was a predictor of consulting behaviour in patients with knee pain; however, this study recruited from only two general practices in London and had relatively low numbers.

In summary, in terms of enabling factors, few studies have evaluated the impact of deprivation on consulting and none have looked at the influence of social networks. Living alone appears to be associated with higher consultation frequency. The influence of previous use of healthcare is an area where conflicting findings exist between quantitative and qualitative research, the former suggesting a positive influence.

### Need factors

#### Severity of pain

Studies show that pain severity is higher in consulters compared to non-consulters [2,5,10,16] in addition to clinically detectable joint swelling [16]. Patients have identified severity of pain as an important trigger to consultation [18,23]. However when severity of pain is included in statistical models to evaluate predictors of consulting, the results are conflicting and appear to be dependent on the tool used to measure pain. Studies that evaluated pain severity using the WOMAC (Western Ontario and McMaster Universities OA Index) indicate that it is not a significant predictor of consulting [10,16], whereas studies using other measures found a significant association [2,17].

The data on consultation frequency would suggest that a large proportion of patients with severe pain are not consulting their GP about joint pain but are consulting with other problems [2]; consulters and non-consulters with severe knee pain had a higher number of co-morbid consultations than those with mild pain. This observation led the authors to suggest that there may be multiple occasions on which to opportunistically assess and manage joint pain when there is another reason for consultation. However, it is possible that discussions regarding joint pain are occurring but are not being recorded, as suggested by Cronan et al. [11].

#### Duration of pain

Recent onset of pain (within one year) has been significantly associated with consulting with knee pain [2]. In contrast, a large postal survey of adults over 50 with self-reported knee pain identified a higher frequency of self-reported consultation rates in those with chronic pain, although in this study chronicity was defined as more than 3 months [5]. It may be that the peak duration of symptoms to trigger consulting is somewhere between three and 12 months.

Characteristics of the pain, such as being of sudden onset, may lead patients to identify symptoms they perceive as less likely to be 'ageing related’ or normal for them and therefore more in need of medical attention [24].

#### Joint affected

Linsell et al. [6] compared the likelihood of consulting in individuals with hip or knee pain. They reported that patients with knee pain were more likely to consult the GP (self-reported rates) than those with hip pain when adjusted for age, sex, severity, bilaterality and duration. Watts et al. [25] report that hand pain was more often referred to as normal for ageing (by patients) when compared with pain at other sites.

#### Disruption of daily activities

Disruption of normal activities appears to be clearly related to consulting behaviours. Mobility problems were the most significant predictor of consulting a GP in a study of patients with self-reported hip and knee pain [17]. The extent to which pain disrupted everyday life was also the most important determinant of the patient’s decision to consult in a conjoint analysis study [20]. Furthermore, activity limitation was also a significant factor affecting consultation rates in a US study of patients aged over 60 with OA [11].

#### Multi-morbidity

OA patients have more multi-morbidity than age and sex matched controls [26]; however how the presence of co-morbid conditions affects consultation remains unclear. Thorstensson et al. [17] found that the number of comorbid conditions was not related to consulting rates in patients with self-reported hip and knee pain in patients aged 35 and over. Bedson et al. [2] also reported that there was no difference in the number of co-morbid consultations in consulters and non-consulters. However, selection bias may have resulted in under-representation of patients with comorbidity, and the Thorstensson study may have included patients who did not have OA due to the age inclusion range.

In contrast, Schellevis et al. [27] report a study from the Netherlands, recording consultation frequency in patients with five chronic diseases, and report that patients with OA are more likely to consult their GP if they have comorbidities compared to single disease (6.4 consultations per year compared with 4.2). However, whether or not the consultation was for joint pain was not recorded and so this finding may be explained by the observation that patients with more severe pain visit their GP more, although not necessarily about their joints [2,21]. This study is limited by missing data in 30% of consultations and only 80 of the total 962 patients had OA of the knee and hip, with other types of OA excluded.

Bedson et al. [2] report that participants’ rating of knee pain as the 'most important health problem’ was significantly associated with likelihood of consulting with knee pain, suggesting that patients do prioritize their health problems. The authors suggest that if comorbid illness is perceived as important this may result in non-consultation for joint related problems.

In summary, disrupted function is a clear influence on consulting. Characteristics of joint pain including severity, duration and distribution also appear to influence consulting decision making. Multi-morbidity appears not to be associated with increased frequency of consultation for joint pain in patients with OA; however this finding may be limited by under-representation of patients in studies or by the completeness of medical record data.

## Conclusions

We have reported the influences on consulting a General Practitioner with OA using Andersen and Newman’s model of healthcare utilisation which incorporates biological, psychological and social factors.

Health beliefs appear to be important predisposing factors in deciding whether or not to seek health care. The belief that OA is an inevitable part of ageing, about which little effective treatment exists and a perceived negative attitude of the GP are reported as disincentives to consulting. Health beliefs are also likely to interact with other identified themes; for example age, and the influence of previous healthcare use on consulting. Previous healthcare use has been associated with increased consulting, but could also result in fewer subsequent consultations if the patient perceived a negative response from the healthcare practitioner consulted. Other important health beliefs include perceiving severe consequences of pain and frustration, which are associated with increased likelihood of consulting. Depression is a further psychological variable for which the evidence is contradictory, and which is likely to be closely related to social context.

The 'need’ factors, in the context of OA are mostly represented by joint related symptoms, impact of the symptoms or comorbidities. Disruption of daily activities appears to be an important driver to seeking medical help. Severity of pain is higher in consulters compared to non-consulters although tests of statistical significance yield contradictory results; individual patients have reported pain as of importance in qualitative research and the lack of statistical evidence to support this may be related to limits of the quantitative measures used. Qualitative research has demonstrated a vast range of descriptors that patients use to describe pain which suggests the questionnaire tools used may be limited in ability to capture the full pain experience [28], which may explain the discrepancy in findings. Again, need factors are likely to interact with an individual’s health beliefs. The physical factors such as severity, distribution and duration of pain may form a 'pattern’ of pain that patients perceive as normal or abnormal, which in turn will influence decision making to seek healthcare.

Patients with OA who consult their GP appear to have more comorbid conditions but how comorbidity affects consulting frequency about joint pain is not clear. Related to this is the finding that patients with severe pain are visiting their GP frequently about issues other than their joints. The literature would suggest these patients are not having their symptomatic joint pain managed, but this may be due to limitations in the various methods of estimating consultation frequency and content. Furthermore, the ways in which patients and doctors prioritise symptoms in the context of multi-morbidity is not well characterised in the literature.

In general, the social aspects and 'enabling’ factors are reported on less frequently than other variables in research in this area, although living alone and the area of GP practice appear to be important. The healthcare system is a further important contextual influence and some of the observed differences in findings may be explained by variation in healthcare access and availability, for example, the relationship between health insurance and financial status and consulting. Furthermore, differences in GP training across countries may impact on the consulting behaviours.

The predisposing, enabling and need factors are not mutually exclusive and there is some overlap between categories. For example, co-morbidities may be 'pre-disposing’ in the case of long term conditions that existed prior to the current illness, or 'need’ factors that are directly influencing the need for seeking health care. A further example are health beliefs, which may be classified as 'predisposing’, 'need’ or 'enabling’ factors. The model has been criticised for generally underplaying psychological factors [10].

An alternative theoretical lens through which to consider access to healthcare is the notion of 'candidacy’ [29], although this was not described in any of the papers in this review. A 'candidacy’ perspective suggests that an individual’s identification of his/her 'candidacy’ for healthcare may be constructed through their economic position as well as by dominant cultural values towards service use which may either increase or curtail health service use. It offers an alternative way of identifying what types of factors make patients 'better’ or 'worse’ candidates for consulting the GP’; based on their own lay beliefs about OA and wider cultural discourses around pain and disability, and their relationship to issues of fairness, equity and 'deservedness’. Candidacy draws together many of the factors discussed in this review, and is also a dynamic process, and this may go some way in explaining why the existing literature does not appear overly sophisticated in its ability to explain consulting behaviour.

One of the general methodological limitations of the studies included relates to estimates of consultation frequency. Consultation prevalence that is calculated using only diagnostic codes may underestimate consultation prevalence as there is evidence that GPs exercise caution when using diagnostic codes and may favour symptom descriptors [30]. Coded data may also underestimate frequencies if not all aspects of the consultation are recorded [31]. However, studies that identify consulters on the basis of all joint related medical record codes as well as a free text search may overestimate consultation frequency of OA specifically as alternative diagnoses will be included. Furthermore, overestimation of consultation rates in some of these studies may be attributable to selection bias due to the possibility that similar factors influence participation in research as those influencing decision-making to seek healthcare. Self-report is limited by recall bias which may over- or under-estimate consultation frequency.

A further limitation of the included studies that used quantitative measures to calculate influences on consulting are that these may underplay the interaction between variables. Depression for example, is an example where the evidence was weak and there may be variation in how this factor could influence consulting. One could argue that depression could both increase or decrease consultation frequency due to coping difficulties and lack of social support or due to isolation. Qualitative research may be better placed to explore complex influences, taking into account the social and environmental context.

This review highlights two particular aspects of the consultation itself that require further evaluation and research. Pain and disrupted activities appear to be important drivers to consulting and so there is therefore a need to establish how these issues are addressed within the consultation. Not all aspects of the consultation may be recorded or remembered and therefore observation research is well placed to explore this further. Secondly, perceived 'negative’ attitudes to osteoarthritis have emerged as disincentives to consulting and a further need exists to establish whether this is evident in consultations. The subjective issue of negativity is also a difficult topic to research using retrospective measures such as post-consultation interviews, and would require a research approach that incorporated multiple perspectives on the consultation.

## Abbreviations

GP: General practitioner; JP: Joint pain; OA: Osteoarthritis.

## Competing interests

The authors have no competing interests.

## Authors’ contributions

ZP conceived the review, conducted literature searches, conducted the narrative review and drafted the manuscript. TS and ABH contributed to the narrative review and the manuscript. All authors read and approved the final manuscript.

## Authors’ information

ZP is a Clinical Lecturer and Honorary Consultant Rheumatologist and conducted this review as part of a PhD using mixed methods to explore the osteoarthritis consultation in primary care. This is part of a larger programme of work aiming to enhance the care of osteoarthritis in primary care. TS is a Senior Research fellow with an interest in consultation research, and supervisor to ZP. ABH is a Professor of Medical Education and Consultant Rheumatologist and PhD supervisor to ZP.

## Pre-publication history

The pre-publication history for this paper can be accessed here:

http://www.biomedcentral.com/1471-2296/14/195/prepub
